# Addressing physician stress, burnout, and compassion fatigue: the time has come

**DOI:** 10.1186/2045-4015-2-32

**Published:** 2013-08-15

**Authors:** Alan H Rosenstein

**Affiliations:** 1 Clinical Efficiency and Care Management, ValleyCare Hospital, Pleasanton, USA

## Abstract

Stress, burnout, and compassion fatigue can have a significant adverse effect of physician well being and patient care. While the frequency and intensity of these negative influences appear to be increasing, there is little help available. We need to raise physician awareness as to the seriousness of this issue and at the same time gain a better understanding of some of the causative factors so we can provide the necessary support services that will enable our physicians to better adjust to the pressures and stresses of our health care environment and re-energize their zest and idealism for medical care.

This is a commentary on http://www.ijhpr.org/content/2/1/31.

## Commentary

Compassion fatigue, burnout and low levels of compassion satisfaction are becoming increasingly prevalent in both the physician and nursing professions [1]. There are a multitude of internal and external factors contributing to these problems, including the contributions of age (generational values), gender, culture and ethnicity, training, personal ideology, and new environmental pressures impacted by advanced technologies, increasing complexity, data availability, cost control, and growing overall performance accountability which are changing clinician and patient expectations of care. This is particularly true in the United States where there is an over arching concern about the significant dollars being spent on health care (over 15% of the Gross National Product) and the less than optimal outcomes achieved (not in the top 20) compared to other nations providing better outcomes at lower costs. As a result health care payers are putting increasing pressures on providers to demonstrate their ability to deliver more appropriate, effective, safe, high quality care. In fact, one of the key goals of the current U.S. health care reform is to develop initiatives designed to reduce unnecessary variation and costs of care. This is being achieved by holding providers more accountable for their outcomes by financially rewarding them for better quality care and/or financially penalizing them for poor outcomes of care. In this regard, many provider organizations have changed their modes and models of care which directly affects the way clinicians practice medicine. All of these factors have contributed to high levels of stress and burnout which have had a significant impact on physician attitudes and behaviors affecting their perspectives and outcomes of medical practice [2].

The recent IJHPR article by Elbar et al. shows a disconcertingly high rate of risk for compassion fatigue and burn out, and low rates of compassion satisfaction for a group of family practitioners working in the Negev region of Israel. Contributing factors include immigration to Israel, female gender, personal life experiences, patient relationships, work-life balance, lack of academic affiliation, and other personal and organizational factors. Suggestions for improvement included education, investigation of contributing organizational factors, and an early intervention treatment program emphasizing well being and resiliency building. The paper goes into an in-depth definition and description of each of these characteristics which can all lead to problems associated with depersonalization and suboptimal patient care [3].

This study highlights the growing problem of stress, burnout, and loss of compassion in our physician population. While some of the causative factors in this study may be more unique for this study population, many of the underlying causes are ubiquitous. Following up on the author’s recommendations for education and intervention, there are many things we can do to help our physician population [4].

First we must recognize physicians as being a precious resource and try to gain a better understanding of their issues so we can help. While some of the ingrained contributing factors such as age, gender, training, and the cultural and life experiences molding personal values, perceptions and behaviors may be difficult to address, many of the external pressures are more amenable to modification.

One of the approaches we have taken at our organization is to orient the physician through a comprehensive on-boarding process. We meet with the physicians, welcome them, and thank them for their contributions. We then discuss any relevant issues that may impact their practice and offer to help them in any way we can. Some of these issues may relate to operational issues such as scheduling, administrative or clerical support, financial support, or advice on practice management, and some of these issues may relate to behavioral attitudes that may benefit from guidance on time management, stress management, conflict management, and/or improving communication and relationship skills.

Increasing the physician’s awareness of the symptoms and problems related to stress, burnout, and compassion fatigue and its negative effect on both their well being and patient care is often the first step toward improvement. After raising awareness the second step requires addressing the reluctance of the physician to admit that it’s true and be willing to accept outside advice or assistance. Many physicians feel that they are used to working under stress and think they have it within themselves to self correct. On top of this is a very strong ego and admitting that they are under stress is a blow to their self esteem. Another contributing factor is the perception that why should they accept advice from outsiders who really don’t understand their world. There is also the fear about confidentiality and the concern about what others may think about clinical competency. Ideally, physicians should take action on their own but in the majority of cases it is the organization that makes the recommendation for next steps.

Organizations need to be sensitive to these issues by assuring confidentiality and reaffirming the goal of providing services that will help the physician practice in a more effective and satisfying manner. These types of services may be afforded through Human Resources, Medical Staff Services, Wellness Committees, a Physician EAP (Employee Assistance Program), or through outside resources. More deep seated issues may benefit from individual coaching or counseling. These types of sessions need to be conducted by trained individuals who are very familiar with the physician’s world. Continued emphasis on the importance of maintaining a positive lifestyle and work life balance should also be stressed and the organization should provide services and logistical support by addressing physician access and convenience and encouraging physicians to make this a priority. As the author’s mention, early intervention is the key. Addressing potentially worrisome behavioral issues early on in the process has a much greater potential for long term success than intervening once a major incident has occurred [5].

Physicians are not the only group which could benefit from these types of services or interventions. Changing technology, increasing complexity, staffing issues, and a growing number of non- clinical administrative tasks are changing the roles and responsibilities in nursing and other health care disciplines. These factors have significantly increased levels of dissatisfaction, burnout, and compassion fatigue to the point that it has negatively affected morale and turnover and led to compromises in patient care delivery [6].

In the end it’s important for organizations to realize the frequency and significance of stress and burnout and be able to work with physicians and other health care disciplines to help them better adjust to their environment. In today’s complex medical environment, we all need to work together as a well functioning integrated team in order to provide best patient outcomes of care. For physicians, we just can’t leave it up to the physicians themselves to take action. They are overworked, time inhibited, trying to juggle multiple issues at one time as part of their dedication to patient care. While they recognize the need for relaxation, self care, and behavioral adjustments other priorities interfere and it’s often pushed to the bottom of the list. We need to re-energize their idealism and zest for being a physician.

## Competing interests

The author declares that he has no competing interests.

## Authors' information

Alan H. Rosenstein M.D. M.B.A. is a practicing Internist in San Francisco, CA, Medical Director of Clinical Efficiency and Care Management at ValleyCare Hospital in Pleasanton, CA, Medical Director of Physician Wellness Services, and a consultant in health care organizational and behavioral management.

## Commentary on

Elbar, N., Levy, A., Wald, H. Biderman, A. “Compassion Fatigue, Burnout and Compassion Satisfaction among Family Practitioners in the Negev area: A Cross Sectional Study. *Isr J of Health Policy Res* 2013, 2:31.

## References

[B1] Shanafelt T Sonja S Tan L Dyrbye L Burnout and satisfaction with work-life balance among US physicians relative to the general US population Arch Intern Med 2012 172 18 1377 1385 10.1001/archinternmed.2012.3199 22911330

[B2] Rosenstein A Physician stress and burnout: what can we do? Am Coll Phys Executives 2012 38 6 22 30 23888648

[B3] Elbar N Levy A Wald H Biderman A Compassion fatigue, burnout and compassion satisfaction among family practitioners in the Negev area: a cross sectional study Isr J of Health Policy Res 2013 2 31 10.1186/2045-4015-2-31 23947591 PMC3765463

[B4] Danielson D Ketterling R Rosenstein A “M.D. Physician stress and burnout: causes, effects, and impact on performance and behavior” AMGA Group Practice Journal 2013 62 No.3 38 41

[B5] Rosenstein A Early intervention can help prevent disruptive behavior Am Coll Phys Executives J 2009 35 6 14 15 19999687

[B6] Aiken L Clarke S Sloane D Sochaiski J Silber J Hospital nurse staffing and patient mortality, nurse burnout, and job satisfaction JAMA 2002 288 18 1987 1993 12387650 10.1001/jama.288.16.1987

